# Xe Recovery from Nuclear Power Plants Off-Gas Streams: Molecular Simulations of Gas Permeation through DD3R Zeolite Membrane

**DOI:** 10.3390/membranes13090768

**Published:** 2023-08-30

**Authors:** Bandar J. Bashmmakh, Xiaoyu Wang, Cynthia J. Jameson, Sohail Murad

**Affiliations:** 1 Department of Chemical and Biological Engineering, Illinois Institute of Technology, Chicago, IL 60616, USA; bbashmmakh@hawk.iit.edu; 2 Separation Science Group, Argonne National Laboratory, Lemont, IL 60439, USA; xiaoyu.wang@anl.gov; 3 Department of Chemistry, University of Illinois Chicago, Chicago, IL 60607, USA; cjjames@uic.edu

**Keywords:** gas separation, zeolite, membrane-based separation, molecular dynamics simulations

## Abstract

Recent experimental work has shown zeolite membrane-based separation as a promising potential technology for Kr/Xe gas mixtures due to its much lower energy requirements in comparison to cryogenic distillation, the conventional separation method for such mixtures. Such a separation is also economically rewarding because Xe is in high demand, as a valuable product for many applications/processes. In this work, we have used Molecular Dynamics (MD) simulations to study the effects of different conditions, i.e., temperature, pressure, and gas feed composition, on Kr/Xe separation performance via DD3R zeolite membranes. We provide a comprehensive study of the permeation of the different gas species, density profiles, and diffusion coefficients. Molecular simulations show that if the feed is changed from pure Kr/Xe to an equimolar mixture, the Kr/Xe separation factor increases, which agrees with experiments. In addition, when Ar is introduced as a sweep gas, the adsorption of both Kr and Xe increases, while the permeation of pure Kr increases. A similar behavior is observed with equimolar mixtures of Kr/Xe with Ar as the sweep gas. High-separation Kr/Xe selectivity is observed at 50 atm and 425 K but with low total permeation rates. Changing pressure and temperature are found to have profound effects on optimizing the separation selectivity and the permeation throughput.

## 1. Introduction

Because of the high energy requirement associated with Kr/Xe separation via conventional cryogenic distillations, researchers have been experimenting with and screening multiple types of adsorbent materials as alternatives. Conventional nuclear fission practices involve releasing these off-gases to the atmosphere at dilute conditions [1]. Kr/Xe gas adsorption has been reported on shales, graphite, silica gel, molecular sieves, activated charcoal, MOFs, and zeolites [2,3,4,5,6,7,8,9]. The large number of feasible adsorbents is due to the fact that both Kr and Xe have similar atomic attributes, including a fully occupied outer electron shell and the fact that both are inert gases. The major difference between the two atoms is in the kinetic diameter (d(Kr) = 3.69 Å and d(Xe) = 4.05 Å), where this difference allows for separation based on size exclusion given adsorbent material cage openings that have suitable dimensions. In this case, for size exclusion separation to have the desired separation factors for Kr/Xe, the rigidity of the adsorbent material framework is crucial to attain high separation factors, which is a feature of many all-silica zeolites, more so than other types of adsorbents such as MOFs, which can be very flexible.

When thinking of membrane adsorption separation, one needs to consider two types of selectivities: First, adsorption selectivity, which is governed by the intermolecular interaction between the adsorbent surface and the guest gas atoms. Second, gas permeation selectivity, which is mainly affected by how easily gas atoms can pass through the membrane mainly due to the smaller atom size. Therefore, an adsorbent must exploit these two characteristics to attain high separation factors for separations of species with similar properties, such as Kr and Xe. Linde Type A (NaA) zeolite has exhibited an adsorption selectivity of 4.1 in favor of Xe over Kr at 1 atm, 300 K, and equimolar gas conditions [6]. Another zeolite, chabazite (CHA), was tested for Kr/Xe gas permeation selectivity, in which separation factors came out as high as 51 in favor of the smaller Kr atom [9].

Experimental work on Kr/Xe separations using the deca-dodecasil-rhombohedral (DD3R) all-silica framework has been carried out with promising separation factors [10]. DD3R membranes were fabricated for these experiments on four-channel hollow fibers with an approximate thickness of 4.4 µm. The membranes prepared by hydrothermal synthesis for 48 h were robust and defect-free. Additional details about the experimental work on such separations are discussed in an earlier paper [8]. The permeation of pure and mixed gases was evaluated via the Wicke–Kallenbach technique, which has been used for other gas permeation performance experiments [11,12]. In this technique, a Ledamass quadrupole mass analyzer is used to measure partial pressures at the feed, retentate, and permeate regions. Such gas permeation experiments use a sweep gas, such as He or Ar, to maintain flow rates and total pressures at the feed and the permeate sides of the membrane.

The present work is an extension of a previous paper published by our group [13], in which we study Kr/Xe gas permeation through a DD3R zeolite membrane, using molecular dynamics simulations to provide an understanding of the experimental trends and ratios of Kr/Xe reported in the experimental literature. In that paper, we showed how our nonequilibrium molecular dynamics simulations of Kr/Xe gas permeation through DD3R zeolite membrane were sufficient to reproduce all experimental trends reported on such separations. The nonequilibrium MD simulations used in our previous work and partially in this one have been implemented successfully to study mass transfer through porous materials in multiple applications, such as alcohol dehydration, ion exchange, gas separations, and reverse osmosis [14,15,16,17]. Furthermore, we have provided an understanding of the contrary permeation trends observed in experimental work between Kr/Xe pure and mixture gases based on molecular observations. We show that the attractive forces between gas particles in the adsorbed phase and in the bulk phase play an important role in the gas permeation rates. Therefore, we ensure equilibrium adsorption conditions within the membrane region of the simulation box in this work, when preparing the initial molecular configurations for gas permeation MD simulations. Here, we revisit the Kr/Xe separation via the DD3R zeolite membrane, but under quasi-steady-state conditions, which we achieve using Grand Canonical Monte Carlo simulations to populate the membrane cavities with an equilibrium occupancy of guest atoms ahead of starting MD simulations.

As explained earlier, experimental work for studying Kr/Xe gas separations in DD3R zeolite use Ar as the sweep gas to promote gas permeation [9]. Unlike our previous paper, which considers only binary mixtures of Kr/Xe, the present work takes this fact into consideration by examining the changes that occur when a ternary mixture is undergoing the gas separation process.

## 2. Materials and Methods

To study quasi-steady-state gas permeation through porous material membranes using molecular dynamics, the initial configuration of MD simulations should reflect the equilibrium adsorption occupancies in the interior cages of the DD3R zeolite membrane. This is important to overcome the short time scales of MD simulation, which would otherwise involve unreasonably long simulation times to achieve equilibrium occupancies in the interior cages of the membrane slab. This approach has been implemented for studying the behavior of the adsorbed water in MOFs under different conditions [18]. In addition, computations of molecular self-diffusivities in porous materials also require the generation of initial configurations that are reflective of adsorption equilibrium states [19]. In our previous paper, we have shown that even manually pre-filling interior alpha cages of DD3R zeolite with the maximum Xe occupancy observed experimentally (2 atoms) was helpful in reproducing experimental Kr/Xe gas mixture permeation trends [13]. In the present work, we generate the initial configurations for the gas permeation simulations using a preliminary stage of the Grand Canonical Monte Carlo (GCMC) process to introduce equilibrium adsorption into the inner cages prior to the start of the MD runs.

**Figure 1:**
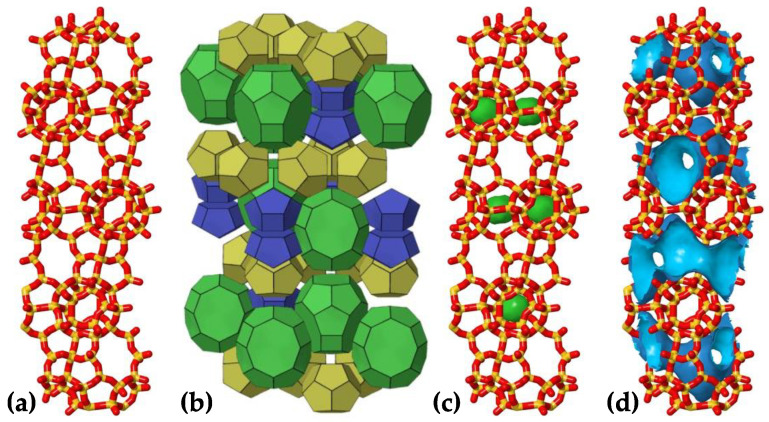
DDR zeolite framework topology. (**a**) bond representation of the DD3R unitcell, (**b**) tiling scheme of the three types of cages in DD3R, (**c**) blocked sodalite cages during GCMC runs, and (**d**) accessible channel system for guest gas atoms. Visualization was done using JMOL extension in the database of zeolite structures [20,21].

**Figure 2:**
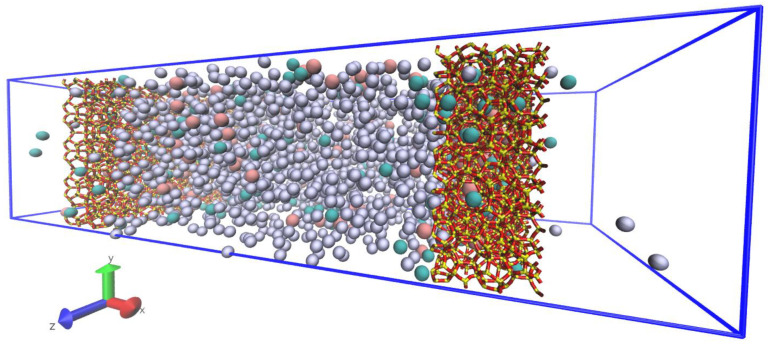
A 3D schematic of the simulation system (222 Å, 55.4 Å and 81.8 Å in the x,y,z directions respectively) Periodic boundary condition extends the system in all three directions. Color code: Kr (cyan), Xe (pink), Ar (violet), Si (yellow), O (red).

**Figure 3:**
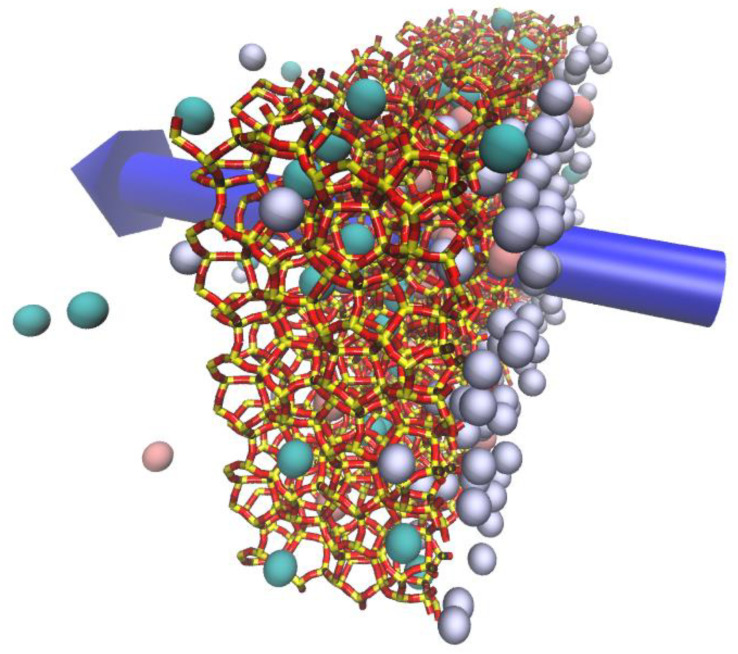
A 3D schematic of the DD3R membrane slab showing the direction of gas permeation from the high pressure to the low pressure regions of the simulation box.

The atomistic unit cell model of the all-silica deca-dodecasil 3R (DD3R) was obtained from the Database of Zeolite Structures [20]. Shown in Figure 1a are the topological details of the DD3R framework code and the tiling patterns of the distinct cages assembling the framework. The unit cell is trigonal with dimensions of 13.795 Å in *a* and *b* directions and 40.750 Å in the ***c*** direction, while the values are 90°, 90°, and 120° for the *α*, *β*, and *γ* angles, respectively. This zeolite framework consists of three types of distinct cages color-coded in Figure 1b. Not all cages undergo gas permeation or adsorption. Only green-coded alpha cages allow for the occupancy and passing of guest gas atoms. Yellow sodalite cages and smaller blue cages do not have suitable cage openings to permit the entry of gas atoms. To quantitatively study gas permeation using MD, a large enough surface must be cleaved from the zeolite into a slab that is periodic and has enough adsorption sites on the surface in contact with the permeating gas atoms. BIOVIA Material Studio provides a user-friendly interface for the manipulation of crystal structures by allowing one to choose the Miller indices for the cleaving plane in addition to automatically taking care of periodicity concerns. The cleaved DD3R slab is modeled after a previous of work of our group on CO_2_/Xe separation via DD3R zeolite [8]. The complete simulation system is shown schematically in Figure 2. The rectangular slab that constitutes the membrane, shown in Figure 3, is 25.2 Å in the direction of gas permeation, with a height of 55.4 Å and 81.8 Å wide. We also investigated the effects of changing the thickness of the membrane while keeping other variables fixed. The number of molecules adsorbed increased as expected when the thickness was increased. However, as expected, the permeation rate decreased since the driving force per unit thickness of the membrane decreased. We truncated the membrane structure in a way that leaves partially open cages that end with the eight-member ring aperture, which acts as an active adsorption site on the surface in contact with the permeating gas. This is important for the DD3R framework to keep the correct chemical formula of the all-silica zeolite by only exposing oxygen atoms during the surface cleaving process.

The zeolite membrane is rigid in both GCMC and MD simulations. We have used the Large-scale Atomic/Molecular Massively Parallel Simulator software package (LAMMPS) to conduct both types of simulations [22]. A consistent 12-6 Lennard–Jones potential was used for all simulations with a global cutoff distance of 12 Å. The functional form of the potential model is given by Equation (1), describing the intermolecular interactions with ε as the depth of the potential energy well, and σ as the interatomic distance at which the potential function is equal to zero. $r_{ij}$ is the pair-wise interatomic distance and $r_{c}$ is the cutoff distance for energy calculations. The potential model parameters for the individual atoms are given in Table 1. The cross-terms for the inter-species interactions were calculated using the Lorentz–Berthelot mixing rules given by Equations (2) and (3).

(1) $$V_{ij}=4\varepsilon_{ij}\left[{\left(\frac{\sigma_{ij}}{r_{ij}}\right)}^{12}-{\left(\frac{\sigma_{ij}}{r_{ij}}\right)}^{6}\right]\;r_{ij}<r_{c}\;$$

(2) $$\varepsilon_{ij}=\sqrt{\varepsilon_{i}\varepsilon_{j}}\;$$

(3) $$\sigma_{ij}=\frac{\sigma_{i}+\sigma_{j}}{2}$$

To introduce equilibrium adsorption conditions into the zeolite membrane slab, we have conducted Grand Canonical Monte Carlo (GCMC) cage pre-filling simulations using a simulation box that encloses the DD3R cleaved slab discussed above. The GCMC insertion volume was selected to include only interior full cages in the DD3R slab, excluding partially open cages on the slab’s truncated surface. These simulations use the “fix GCMC” command, which requires the MC package to be installed in the LAMMPS build. This command performs grand canonical Monte Carlo exchanges between an imaginary gas reservoir and the simulation box. The exchanging gas reservoir is defined using atom type, temperature, and pressure, while the fix ensures the chemical potentials of atoms in both the simulation box and in the gas reservoir are equal. In the case of gas mixture simulations, a distinct “fix GCMC” command line is used for each gas species. In such cases, the pressure specified for each gas species is the partial pressure in the gas phase. We ran 10,000 cycles of MC simulation; each step includes 10 GCMC exchanges, which are either insertions or deletions with equal probability, and 10 GCMC moves, with a translational displacement of only 0.1 Å for atomic noble gases. Frenkel and Smit have explained the GCMC exchanges and moves acceptance criterion, and how the pressure of the exchanging reservoir is related to its chemical potential within this command [24]. A specific challenge associated with the DD3R framework is the void made by the small sodalite cage shown in green in Figure 1c, which is large enough to be occupied by gas atoms, and hence it poses a challenge when running GCMC simulations to pre-fill the larger alpha cages. To circumvent this, we have blocked sodalite sites using ghost atoms that make these sites inaccessible to gas atoms.

A range of different temperatures, pressures, and gas phase compositions were considered to prepare the zeolite membrane slabs for gas permeation MD simulations. Additional low-pressure data points were considered while performing the GCMC computations to compare the results with available experimental adsorption data for such gases in similar adsorbents. Furthermore, the GCMC results for pure gas adsorption for Ar, Kr, and Xe in DD3R were fitted using the Langmuir adsorption isotherm model.

To simulate the correct gas densities for the temperature and pressure conditions considered, we ran 1 ns MD simulations of only gas atoms in the isothermal–isobaric (NPT) ensemble. The time integration in these simulations is undertaken using the Nose-Hoover style non-Hamiltonian equations of motion. The timestep used here is 1.0 fs. Thermodynamic output was recorded every 1000 steps after allowing the system to equilibrate for 0.1 ns. The averaged gas densities obtained from NPT simulations were verified using Lee–Kesler generalized-correlation tables [25].

Using the densities from the NPT computations, we have packed the gas permeation initial configurations using the PACKMOL software package, which facilitates the generation of non-overlapping molecular structures [26]. The GCMC pre-filled membrane slabs trap the packed gas atoms in the central region of the simulation box, separating the high-pressure feed region from the low-pressure complete permeation region at the edges of the simulation box, while maintaining the periodic box boundaries in all directions. We note that the two membrane surfaces in contact with the gas feed are not symmetric. This is a result of one slab being only a translated replica of the other without rotation. This setup of the system enables the study of gas permeation through the channel system of the zeolite framework, shown in Figure 1d. Gas permeation MD simulations use the Nose-Hover non-Hamiltonian canonical (NVT) ensemble.

The timestep used is 1.0 fs. An initialization simulation of 0.1 ns to allow the feed gas to equilibrate with the membrane surface is followed by 30 ns of production runs. The quantitative analysis of the permeate, retentate, and feed regions was done by counting gas atoms in each region and the calculation of running averages over the period of trajectory production runs. Density profiles along the gas permeation direction were obtained by chunking the simulation box into 1.0 Å-sized bins, counting the atoms in these bins, and calculating the running averages over the whole trajectory. This is shown in Figure 3, in the membrane slab showing the direction of permeation and how the atoms are counted. We also report effective diffusion coefficients calculated from mean square displacements (MSD) calculated by the native “compute MSD” command in LAMMPS. Visualization of molecular trajectories was undertaken using VMD [27].

## 3. Results

For MD to yield meaningful permeation results, which are the focus of this work, a high driving force is needed to yield a quantitative distinction when studying systems under different conditions, i.e., change of temperature, pressure, and gas composition. This is achieved by considering significantly higher pressures than what would be normally used for studying the same process experimentally; the latter is in the range of atmospheric pressure. In addition, due to the nature of the short time scales achievable in MD, which cause unreasonably long simulation times to be required to reach the adsorption equilibrium conditions in cages of the DD3R membrane, GCMC steps were used to achieve equilibrium cage occupancies ahead of MD runs.

### 3.1. GCMC Adsorption

**Figure 4:**
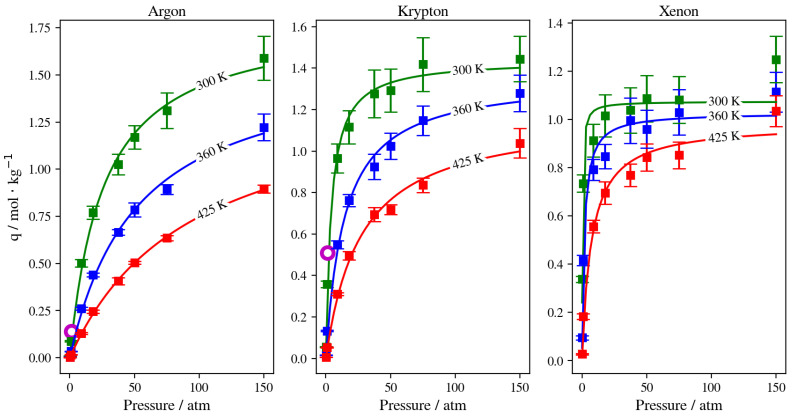
Pure adsorption isotherms for Ar, Kr, and Xe obtained using GCMC simulations and fitted to the Langmuir adsorption isotherm. Experimental data points are visualized by the purple-colored open circle [12].

To provide a reasonable comparison with experimental results on noble gas adsorption in DD3R zeolite, using the “fix GCMC” command, we acquired additional lower pressure data points than would be needed for MD simulations. Each GCMC simulation’s interior data point has been calculated by computing the rolling average of the adsorbed atoms after each system had reached equilibrium. The standard deviation values calculated for each average value are shown in error bars. All results were fitted into the Langmuir adsorption model functional form represented by line plots to compare with experimental results (when available) for the considered gas species’ adsorption in DD3R. Figure 4 shows the adsorption isotherms obtained from GCMC simulations for pure Ar, Kr, and Xe, respectively. As shown, we developed the pure adsorption isotherms of each gas species for the temperatures used in the gas permeation MD runs, which are 300 K, 360 K, and 425 K. This is important when testing for the validity of the “fix GCMC” approach when it relates to higher pressure ranges. Experimental results were available for only Ar and Kr. For both gases, the experimental data points correspond to our results from GCMC simulations. All calculated isotherms have trends that are monotonic as pressure is increased, and plateau when the occupancy of the inner cages of the membrane has reached saturation levels associated with the operating temperature and pressure. Ar adsorption isotherms at the three different temperatures shown in Figure 4 increase as pressure increases, with wide plateaus signifying the higher pressure needed to reach saturation conditions. This is justified because of the smaller Ar atom size in comparison to Kr and Xe, both of which have isotherms that level to saturation conditions at lower numbers of adsorbed atoms than Ar. This effect is also observed, but in a much less visible way, for Kr adsorption isotherms at 360 K and 425 K shown in Figure 4, unlike at 300 K, where Kr adsorption isotherms reach saturation levels at a lower pressure range than for the other two higher temperatures. This provides an insight into the lower saturation occupancy caused by the increase in atom size when switching from Ar to Kr. This effect is even more prominent for Xe, where all three adsorption isotherms shown in Figure 4 reach saturation occupancy, corresponding to a lower number of adsorbed gas atoms, at much lower pressure ranges than the smaller atoms Kr and Ar, respectively. The error bars for all the calculated adsorption isotherms correlate well with the functional form fitting used, another means of confirming the validity of our fit for the limited number of data points from GCMC simulations, which take long simulation times.

As shown by the pure adsorption isotherms, GCMC atom insertion simulations yielded reasonably good adsorption equilibrium results that agree with available experimental results. This is true for pure gases and for Kr or Xe simulations that have Ar as sweep gas at a 10:90 molar ratio. However, in the case of Kr/Xe equimolar mixture simulations, not all GCMC runs of Kr/Xe, either with Ar present or not, reach adsorption equilibrium within the simulation steps considered. While the simulations carried out at high density and low temperature trend in the correct direction toward equilibrium, they would require unrealistically longer simulation times. This is attributed to the insufficient energy possessed by Xe to overcome the insertion barrier. The selected ranges of pressure and temperature for gas permeation MD simulations were identified for simulations that have inner cages at near adsorption equilibrium.

The molecular configurations resulting from GCMC simulations (which include the DD3R slab structure and the adsorbed gas atoms in their favorable positions within the interior cages of DD3R) were used for preparing the MD gas permeation simulation box, as explained above. Using the densities computed from NPT gas simulations, we used PACKMOL to fill the region trapped between the two membranes for the desired gas feed conditions. This way, our MD simulation will capture permeation behavior through the membrane cages while having the adsorption equilibrium conditions satisfied from the beginning of the simulation.

**Figure 5:**
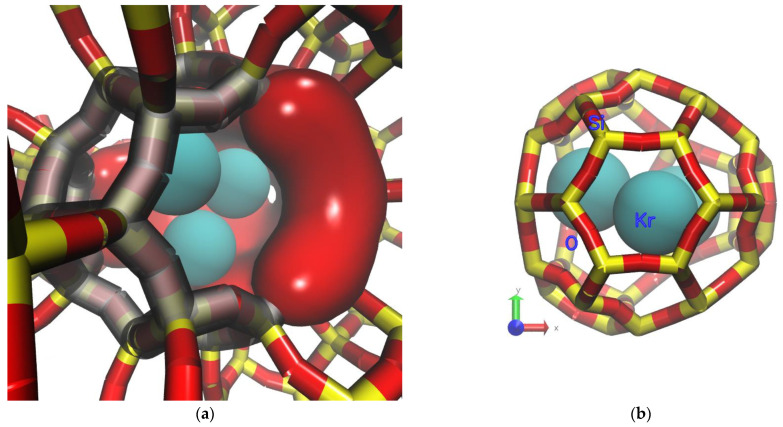
Molecular-level visualization of Kr adsorption behavior in the interior DD3R cage. (**a**) Internal adsorption surface of DD3R alpha cage. (**b**) Example of a favorable adsorption site.

The flow splitting nature of the DD3R alpha cage, in which gas atoms hop into the interior part of the cage via an eight-member ring window and leave the cage via two other similar openings, causes the adsorption surface in the interior of the cage to take the shape of a three-way ring on which atoms are adsorbed, as shown in Figure 5a. The fact of the Si and O atoms forming the surface away from the cage opening results in favorable adsorption sites on the inner surface of the cage. A visualization of such adsorption sites is shown in Figure 5b.

### 3.2. Gas Permeation

Our gas permeation MD runs are divided into two main categories. The first are systems that consist of Kr and Xe pure and equimolar mixtures under different temperatures and pressures. These are similar to the ones we assessed in our previous paper [13]. The difference here is that GCMC adsorption equilibrium conditions are incorporated into the initial molecular configuration. The second category includes simulations of Kr/Xe pure and equimolar mixture conditions with Ar used as the sweep gas at much higher partial pressure than both Kr and Xe. This category considers the same MD simulation scheme for non-equilibrium that we used for gas permeation in the earlier paper, in which Ar reaches equilibrium at much shorter timescales in comparison with both Kr and Xe, due to its small atom size and the lower interaction energy with the zeolite framework (as seen in Table 1).

#### 3.2.1. Pure Gas Krypton

To understand the effects of the different conditions on the equimolar gas permeation, we started with pure gas simulations at similar conditions to isolate the effects of having a binary mixture on the permeation process, as in the previous paper. Pure Kr and pure Xe MD simulations are analyzed for completely permeating atom numbers, fractional permeation, mean squared displacement diffusion coefficient, and density profiles along the direction of permeation, as follows.

**Figure 6:**
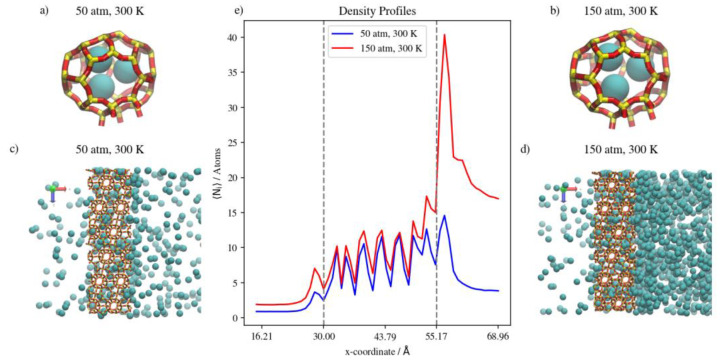
(**a**,**b**) Schematic of an alpha cage in the membrane, (**c**,**d**) an orthographic view of the adsorption layer adjacent to the membrane and (**e**) density profiles of pressure effect on Kr permeation. Conversion factor to density/number = 4378.974 Å^3^ for the density profiles in this figure and subsequent figures.

At a constant temperature, Kr adsorption on the membrane surface (i.e., the surface exposed to the gas feed region and the surface of the inner walls of accessible alpha cages) will increase as pressure increases. This is true as well for the complete permeation of Kr atoms to the low-pressure region at the edges of the simulation box. This is attributed to the permeation process being adsorption-dominant rather than diffusion-dominant. In such cases, the increased adsorption caused by the higher driving force at higher feed pressure, and the limited occupancy of inner cages of the rigid DD3R structure (three atoms for Kr), facilitates higher complete permeation. Figure 6e shows density profiles for Kr. When the pressure is increased from 50 atm to 150 atm at a constant temperature of 300 K, the integral of the peak at the left low-pressure region increases by 72 percent. This is attributed to the effect of the limited occupancy on promoting complete permeation. It is worthy to note that most additional Kr atoms adsorbed are located on the adsorption layer at the surface exposed to the feed gas. This is shown by the higher adsorption peaks of the density profiles at higher pressure in Figure 6e. At 150 atm and 300 K, the adsorption layer peak shows a shoulder forming away from the membrane surface. This agrees with the BET interpretation of monotonic isotherms, as in Figure 4, where only the 150 atm adsorption point is in the linear part of the isotherm, consistent with the saturation of the monolayer. The alpha cage of DD3R is too small to accommodate multi-layer adsorption. In addition, the increase in the integral value between the two pressures inside the membrane is only 26 percent, which shows the dominance of adsorption over the diffusion of pure Kr through the membrane; near-saturation levels are reached in the inner cages independent of pressure. On the other hand, this is not the case for the lower-pressure density profile, where Kr density numbers are not sufficient to accommodate multi-layer adsorption on the surface exposed to the gas feed.

The situation inside the inner cages is different. While the running average of the number of atoms inside the membrane increases with pressure, density profiles are virtually similar within the inner cages of the membrane region. The molecular level visualizations in Figure 6a,b show a limited change in inner cage capacity as pressure increases. Figure 6c,d and the following similar molecular visualizations are orthographic top views of the system along the direction of gas permeation, which shows similar Kr uptake in the membrane region but a higher number of Kr atoms in the complete permeation region at the higher pressure, as well as at the adsorption layer on the surface exposed to the gas.

**Figure 7:**
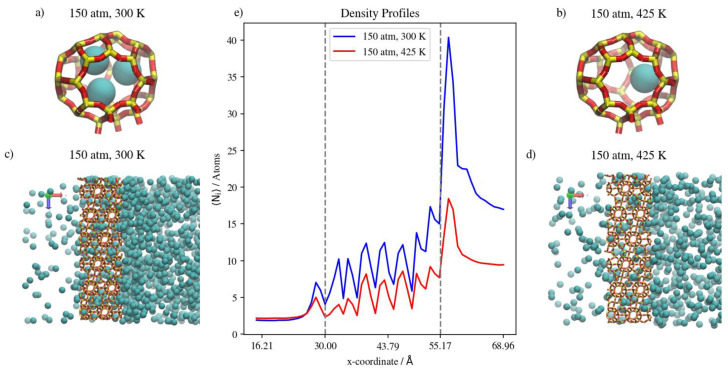
(**a**,**b**) Schematic of an alpha cage in the membrane, (**c**,**d**) an orthographic view of the adsorption layer adjacent to the membrane and (**e**) density profiles of temperature effect on Kr permeation.

**Figure 8:**
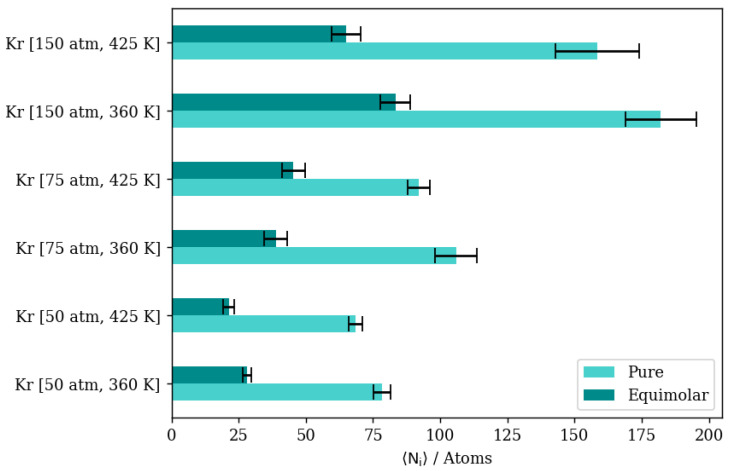
Kr permeance through DD3R zeolite at different temperatures and pressures.

Changing the temperature at a constant pressure, on the other hand, will decrease the amount of adsorbed Kr atoms as well as the number of completely permeating atoms. At 50 atm, increasing the temperature significantly decreases the numbers of adsorbed atoms as well as the numbers that achieve complete permeation. This effect starts to be less obvious when increasing temperature at constant 75 atm (or 150 atm), where the higher density numbers allow for additional adsorption independent of temperature, making density profiles within the membrane much closer to each other than at 50 atm. Complete permeation is also promoted by increasing temperature at higher pressure values, since permeation becomes less sensitive to temperature increases as pressure increases. This is attributed to the high driving force at higher pressures and the higher probability of Kr atoms encountering the zeolite framework than at lower pressure, even as the interaction energy between Kr atoms and the zeolite framework favors adsorption independent of pressure. The occupancies of individual alpha cages taken from molecular trajectories (visualized in Figure 7a,b) were found to decrease when temperature was increased at constant pressure. This is also the case at the membrane surface in contact with the gas feed region, as shown in Figure 7c,d. Figure 7e shows that the density profiles at 150 atm decrease when temperature is increased from 300 K to 425 K in the membrane region, but to a lesser extent than at the adsorption layer. This is not the case in the complete permeation region where density profiles become quite close to each other. This is anecdotal evidence of the ease of the thermal regeneration of the adsorbent, where the rate of desorption becomes higher than the rate of adsorption at elevated temperatures. Therefore, to theoretically increase the permeation throughput, high pressures and low temperatures are required, and this is clearly shown in the summary of pure Kr’s complete permeation results in Figure 8.

#### 3.2.2. Pure Gas Xenon

**Figure 9:**
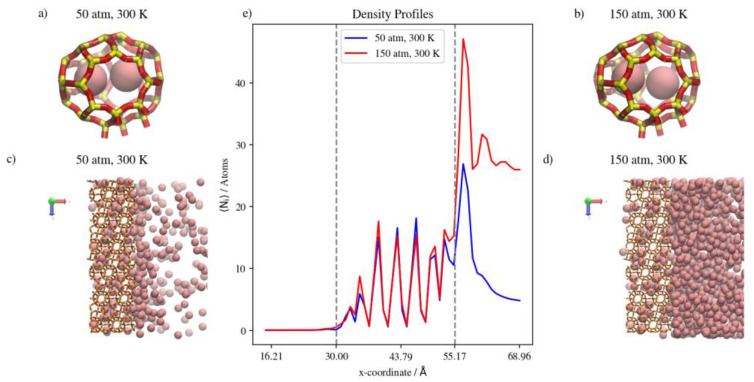
(**a**,**b**) Schematic of an alpha cage in the membrane, (**c**,**d**) an orthographic view of the adsorption layer adjacent to the membrane and (**e**) density profiles of pressure’s effect on Xe permeation.

**Figure 10:**
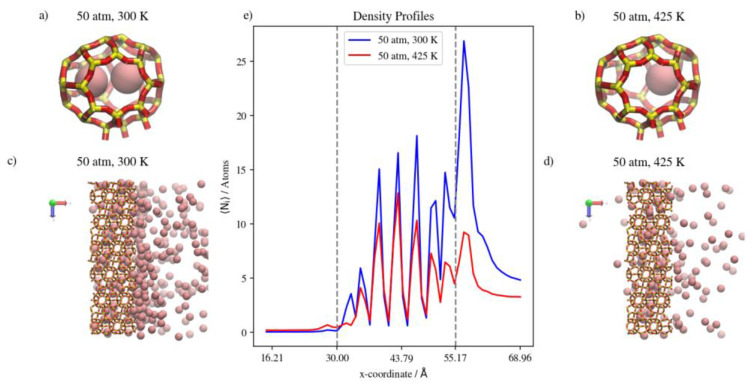
(**a**,**b**) Schematic of an alpha cage in the membrane, (**c**,**d**) an orthographic view of the adsorption layer adjacent to the membrane and (**e**) density profiles of temperature’s effect on Xe permeation.

In the case of pure Xe, we should note that the complete realization of the effects of different conditions on its permeation are difficult, due to the much longer simulation times required to obtain quantitative results. This is attributed to the larger atom size for Xe; its barrier-limited hopping through the eight-member cage openings becomes the permeation rate-determining step. Since this is the case, we are considering changes in the adsorbed phase of Xe on the membrane’s surface to draw our conclusions regarding the different permeation conditions. Increasing the pressure at the constant temperature of 300 K has virtually no effect on complete permeation, and only slightly increases the amount of adsorbed Xe atoms in the membrane region. This was predicted by the pure Xe adsorption isotherm at 300 K, where all three pressure values lay on the saturated linear part of the isotherm, as shown in Figure 4. Similarly, inside the inner cages of the membrane, all density profiles are virtually similar, given the presence of Xe saturation conditions independent of pressure; the Xe occupancies of inner cages remain stable, as shown in Figure 9a,b. This is true for the increasing pressure of Xe at constants of 300 K and 360 K. This effect starts to fade away at 425 K, where increasing pressure from 50 atm to 75 atm slightly increases the amount adsorbed and decreases the complete permeation. More so, increasing the pressure to 150 atm increases the amount of adsorbed Xe atoms as well as completely permeated ones. Both the latter effects were also predicted by the GCMC adsorption isotherm shown in Figure 4, where the 425 K isotherm does not seem to approach pronounced saturation, unlike the Xe isotherms at both 300 K and 360 K. The density profile of pure Xe in the direction normal to the membrane surface forms multiple adsorption peaks at the surface in contact with the gas feed. This is shown by the different saturation levels of the adsorption layer shown for 50 atm and 150 atm in Figure 9c,d. There is no real effect on the density profile in the inner cages of the membrane and the complete permeation regions when pressure is increased from 50 atm to 150 atm at a constant of 300 K, as shown in Figure 9. Increasing temperature from 300 K to 425 K decreases inner cage occupancy as well as the number of Xe atoms at the adsorption layer, as shown by the visualizations from molecular trajectories in Figure 10a–d. The density profiles at the adsorption layer and inside the membrane regions decrease when the temperature is increased at a constant pressure. This is not the case in the complete permeation region, where density profiles increase when temperature is increased, as shown in Figure 10.

This suggests a distinction between pure Kr and pure Xe on the molecular level; that is, the monolayer occupancy is less sensitive in the case of Xe to changes in pressures and temperatures than that of Kr. As shown by density profile calculations, the ratio of the integral over the adsorption layer between 150 atm and 50 atm for Xe is 2.36, while it is 3.25 for Kr, suggesting the reaching of saturation of monolayer occupancy at lower pressures for Xe. This is also shown by the molecular visualization of the membrane surface for both Kr and Xe at different conditions in Figure 6, Figure 7, Figure 9 and Figure 10. The effect of increasing the temperature on Xe’s complete permeation is different than that of Kr’s. Given that both pure gases’ permeations are adsorption-dominant rather than diffusion-dominant, and the limited occupancy of inner cages, increasing temperature increases the complete permeation of Xe atoms. We note, however, that Xe’s hopping into and out of the framework cages is the permeation rate-determining-step, and the complete realization of its permeation behavior would require much longer simulations. In comparison to Kr, the fact that Xe’s maximum occupancy of DD3R alpha cages is two atoms causes this behavior, whereas for Kr, the occupancy of inner cages is not at its maximum of four atoms, causing fewer total Kr atoms to completely permeate. This behavior is expected to change in the case of mixtures, wherein we will be able to study the effects of competitive adsorption.

#### 3.2.3. Equimolar Mixture of Krypton and Xenon

**Figure 11:**
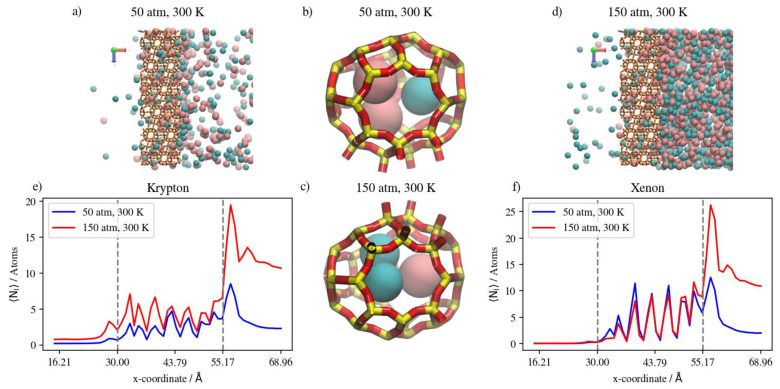
(**a**,**d**) An orthographic view of the adsorption layer adjacent to the membrane, (**b**,**c**) schematic of an alpha cage in the membrane, and (**e**,**f**) density profiles of pressure’s effect on equimolar Kr/Xe gas mixture permeation.

In the case of equimolar Kr/Xe mixtures, where competitive adsorption is in effect, both species’ density profiles do decrease in comparison to pure gases’ density profiles. However, the effects on Xe are much less pronounced than those on Kr. At 50 atm and 300 K, the number of Kr atoms in the membrane region of the simulation box decreases to a third in an equimolar mixture with Xe in comparison to the pure case. This is not the case at the adsorption layer on the surface exposed to the gas feed, where the mixture to pure ratio of the integral values over the adsorption peak is 56.2 percent. This is caused by the fact that we have adsorption equilibrium conditions inside the membrane during GCMC simulations, which are selective toward Xe, as one can infer from the pure adsorption isotherms. Nonetheless, the barrier-height-limited Xe permeation caused by the size of the cage opening (3.6 × 4.4Å) allows for more Kr to accumulate at the surface exposed to bulk gas. With mass transport remaining adsorption-dominant and with the inner cages showing saturation conditions, lower numbers of Kr atoms completely permeate the membrane structure when the feed is an equimolar mixture. Nonetheless, complete permeation selectivity is in favor of Kr over Xe, justifying the candidacy of zeolite membrane adsorption as a more suitable separation unit operation. Increasing pressure at a constant temperature of 300 K would increase both Kr adsorption on the membrane surface and its complete permeation. However, this effect seems to take two different manifestations when pressure is increased to 75 atm or 150 atm. At 75 atm, there is a limited increase of 15.4 percent in the number of Kr atoms in the membrane region, coupled with a two-fold increase in the numbers of Kr that achieve complete permeation. At 150 atm, this effect is different, with a 41.7 percent increase in the number of Kr atoms in the membrane region, coupled with a 54.9 percent increase in complete permeation in comparison to the permeation at 75 atm. This is a direct consequence of the adsorption dominance, where Kr as well as Xe density profiles (shown in Figure 11e,f) show multiple peaks at the surface in contact with feed gas, and the saturation of adsorption sites impedes additional complete permeation. Single cage occupancies are stable for Xe, unlike Kr, which increases in an equimolar Kr/Xe mixture, as shown by molecular visualizations in Figure 11b,c. Visualizations of the adsorption layer at the two different pressures are shown in Figure 11a,d.

**Figure 12:**
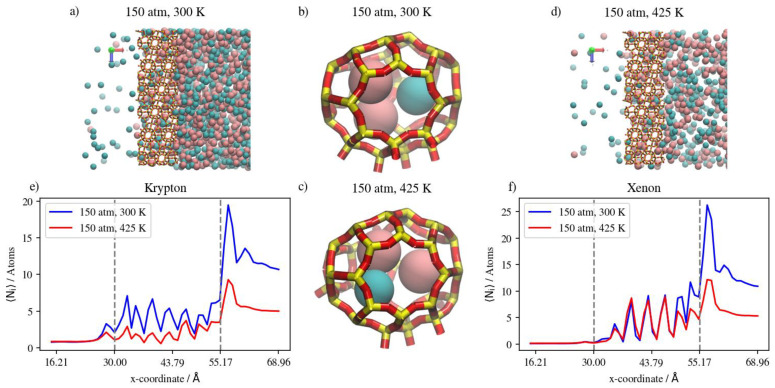
(**a**,**d**) An orthographic view of the adsorption layer adjacent to the membrane, (**b**,**c**) schematic of an alpha cage in the membrane, and (**e**,**f**) density profiles of temperature’s effect on equimolar Kr/Xe gas mixture permeation.

Increasing the temperature at a constant pressure, on the other hand, has diverse effects in an equimolar mixture at the three pressure values of 50 atm, 75 atm, and 150 atm, respectively. At 50 atm and 150 atm, increasing the temperature would decrease the amount of Kr atoms in the membrane region, as well as limiting complete permeation. This effect is attributed to changes in density numbers between the two pressure values. At the lower pressure, increasing the temperature results in low density numbers of Kr, with 254 atoms at 50 atm and 425 K. This would be an insufficient amount to study the effects of temperature on permeation, as predicted by the pure Kr isotherm at elevated temperatures and 50 atm, whereat the conditions are still far from saturation. At the higher pressure, while the same temperature effect prevails on both adsorption and complete permeation, the molecular-level observations shown in Figure 12a,d and the density profiles in Figure 12e,f show that extremely high density numbers at a higher pressure and lower temperature cause the formation of a multi-layer adsorption layer induced by the attractive gas–gas atomic forces in Kr, blocking easy permeation through the membrane structure. Visualizations of the inner cages are shown in Figure 12b,c.

**Figure 13:**
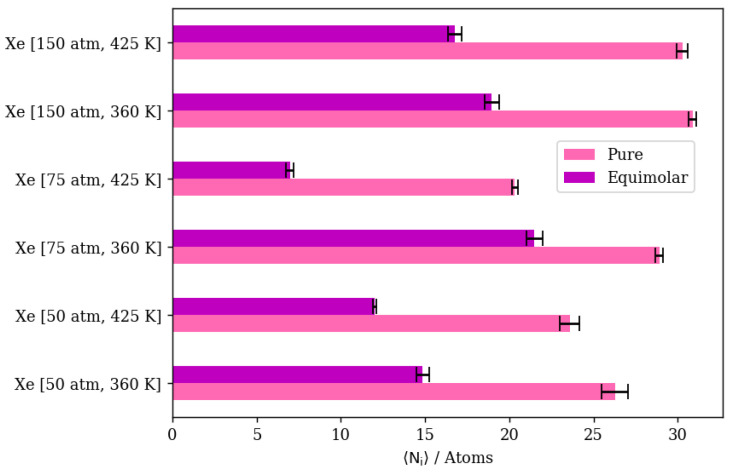
Xe’s permeation through DD3R zeolite at different temperatures and pressures.

This is not the case at 75 atm, where increasing the temperature from 300 K to 360 K would slightly decrease the number of completely permeating Kr atoms while having virtually no effect on fractional permeation, which decreases from 8.60 percent to 8.33 percent. A further increase in temperature to 425 K would reduce the amount of adsorbed Kr but would increase its complete permeation. In this case, we note that it is the highest fractional permeation (of 11.84 ± 1.11%) of all equimolar Kr/Xe simulations. Moreover, with respect to fractional permeation, this is the highest observed, even in comparison to pure Kr simulations, in which the highest fractional permeation is 11.73 ± 0.67%. This is because at 75 atm, the density numbers of Kr provide enough driving force to facilitate permeation without overcrowding the adsorption layer. The overcrowding of the adsorption layer at 150 atm is shown in Figure 12e where the Kr density profile adsorption layer shows multiple peaks away from the membrane surface, signaling the saturation of the monolayer. This is also shown by the Kr/Xe ratio in the membrane region increasing from 0.382 to 0.434 when pressure is increased from 75 atm to 150 atm at 425 K, showing the additional membrane occupancy of Kr with Xe remaining relatively stable; meanwhile, the Kr/Xe ratio within the adsorption layer remains stable. In our previous paper, we have shown that manually filling inner cages with only xenon at its maximum occupancy of two atoms facilitated more Kr permeation in an equimolar mixture in comparison to starting the simulation with empty cages, even in comparison to pure simulations. This is observed in this work at 75 atm, where increasing the temperature would sway adsorption selectivity further in favor of Xe in a way that promotes further complete Kr permeation at adsorption equilibrium conditions in an equimolar mixture. This further suggests that permeation conditions, where saturation levels are achieved for Xe but not for Kr, are preferential for promoting higher separation selectivity. Similar to Figure 8, a summary of Xe’s complete permeation results, i.e., pure vs. equimolar at the different pressures and temperatures, is provided in Figure 13.

#### 3.2.4. Mixtures of Krypton and Argon

As explained earlier, experimental work for such simulations utilizes Ar as the sweep gas to facilitate gas permeance. This would introduce a ternary mixture that undergoes permeation through the zeolite membrane. To understand this, we ran another set of 30 ns MD simulations with Ar at much higher partial pressure than Kr and Xe. All simulations have pre-equilibrated gas atoms in the inner cages of the membrane using GCMC preliminary simulations. In the feed region, the initial number of packed gas atoms includes a 90:10 ratio between Ar, the sweep gas, and Kr/Xe pure or mixed gases. This also enables the examination of separation under dilute conditions for both Kr and Xe. We ran such MD runs for the same range of pressure and temperature as used in the Kr/Xe-only simulations.

In a 10:90 Kr/Ar gas mixture, increasing the pressure at constant temperature would increase the number of atoms of both species in the retentate and the permeate regions of the simulation. At 360 K, increasing the pressure from 50 atm to 75 atm and then to 150 atm has a limited effect on the amount of Kr atoms in the membrane region, due to inner cages reaching near-saturation levels at these conditions. This in turn causes higher pressure to yield higher Kr complete permeation. Increasing the pressure from 50 atm to 150 atm at a constant temperature of 360 K increases Kr complete permeation by 130 percent, while it increases Kr in the membrane region by only 16 percent.

**Figure 14:**
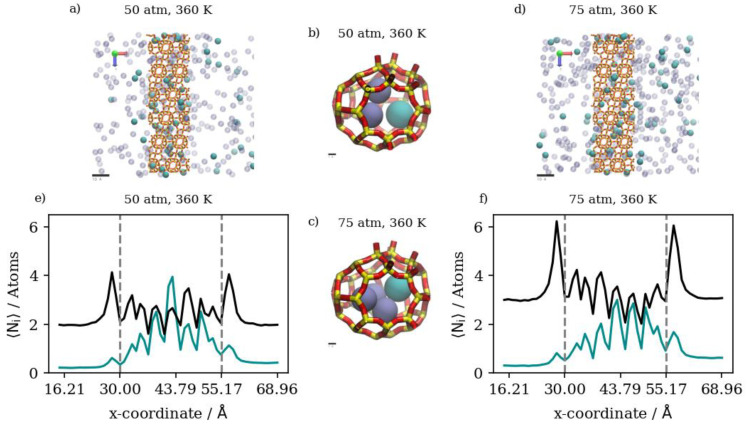
(**a**,**d**) An orthographic view of the adsorption layer adjacent to the membrane, (**b**,**c**) schematic of an alpha cage in the membrane, and (**e**,**f**) density profiles of pressure’s effect on Kr/Ar gas mixture permeation at 360 K. Atoms’ colors: Kr (cyan), Ar (violet). Density profiles’ colors: Kr (cyan), Ar (black).

The molecular-level visualizations in Figure 14a–d show the limited effects that a change in pressure has on the Kr occupancy of different regions of the simulation box. The density profiles in Figure 14e,f show that increasing the pressure only increases the levels of Ar in the membrane region due to the higher density numbers at the higher pressure. For Kr, the integral value of the density profile over the interval corresponding to the membrane region remains constant, independent of pressure increase. On the other hand, with the difference between Kr density profiles at the two different pressures, numerical comparisons between the two cases show almost a 40 percent increase in Kr complete permeation. We note that the distinguished high Kr peak in the membrane region at 50 atm and 360 K is merely a result of the initial configuration from the GCMC simulation not achieving real equilibrium in all the inner cages of the membrane slabs within the MC simulation time considered.

**Figure 15:**
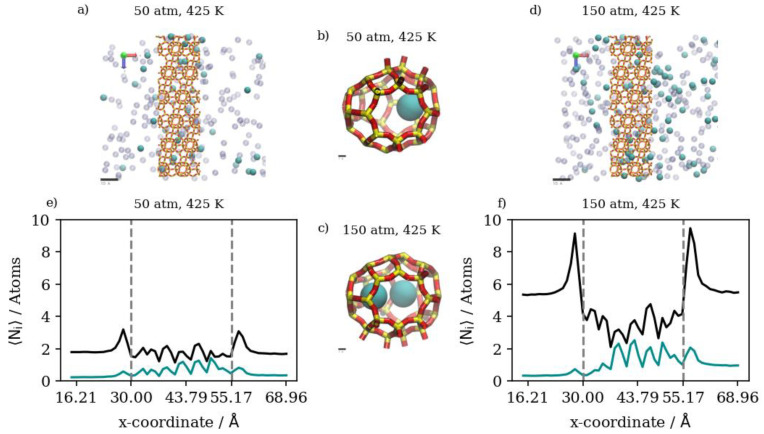
(**a**,**d**) An orthographic view of the adsorption layer adjacent to the membrane, (**b**,**c**) schematic of an alpha cage in the membrane, and (**e**,**f**) density profiles of pressure’s effect on Kr/Ar gas mixture permeation at 425 K. Atoms’ colors: Kr (cyan), Ar (violet). Density profiles’ colors: Kr (cyan), Ar (black).

**Figure 16:**
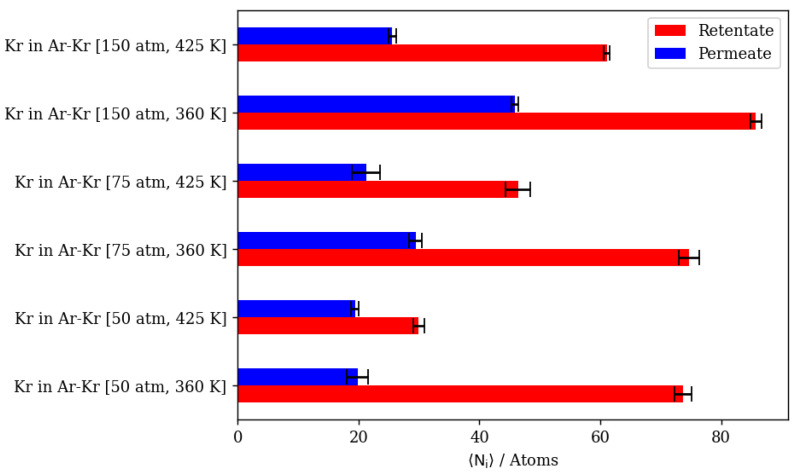
Kr permeance through DD3R zeolite at different temperatures and pressures.

At 425 K, increasing pressure has an obvious effect on the amount of Kr in the membrane region, in which Kr adsorption increases proportionally to the increases in pressure. Complete permeation increases only slightly due to the increased membrane uptake at the higher pressure. This behavior is a result of the extremely low Kr density numbers at the higher temperature combined with the abundance of active adsorption sites, causing Kr atoms to remain inside inner cages rather than passing through. The membrane slabs and inner cages visualized in Figure 15a–d show changes in the occupancy of inner cages when pressure is increased at 425 K. The density profiles in Figure 15e,f show the distinct differences in the Kr occupancy of inner cages due to changes in pressure at higher temperatures. Figure 16 summarizes the numerical results of atom counts in both the retentate and permeate regions of the simulation box for Kr/Ar simulations. As shown in the numerical results, increasing temperature at constant pressure would decrease the number of Kr atoms in both the retentate and permeate regions, further validating adsorption’s dominance over the gas permeation process. In addition, the maximum occupancy of both the adsorption layer and inner cages decreases as temperature increases. The Kr/Ar ratio in the adsorption layer and inside the membrane boundaries shows no significant changes related to temperature increase at a constant pressure.

When temperature is increased, the number of completely permeating Ar atoms decreases more markedly than the number of Kr atoms. Increasing the temperature further to 425 K has no significant effect on Kr’s complete permeation at 50 atm.

**Figure 17:**
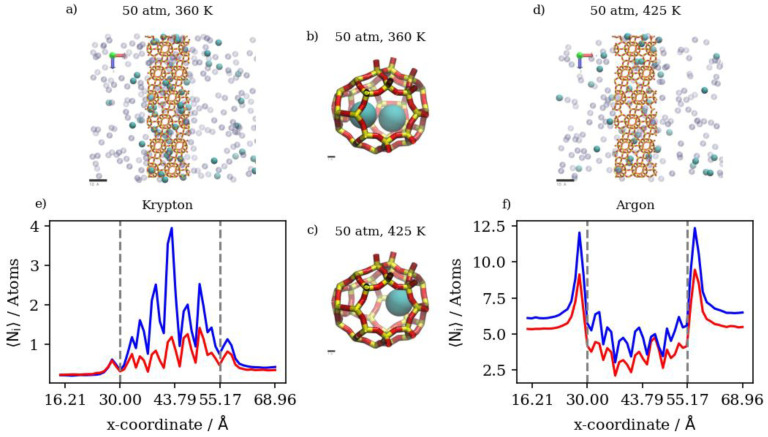
(**a**,**d**) An orthographic view of the adsorption layer adjacent to the membrane, (**b**,**c**) schematic of an alpha cage in the membrane, and (**e**,**f**) density profiles of temperature’s effect on Kr/Ar gas mixture permeation at 50 atm. Atoms’ colors: Kr (cyan), Ar (violet). Density profiles’ colors: 360 K (blue), 425 (red) (see Figure 6 for molecular level picture description).

This is caused by the permeation process being adsorption-dominant; having a lower number density for Kr will result in most of it remaining in the retentate region rather than passing through, due to its higher adsorption selectivity in comparison to Ar. Inside the membrane region, increasing temperature at the same pressure would significantly decrease the amount of Kr. This is not the case for increasing the temperature at a constant pressure value of 75 atm or 150 atm. In such cases, increasing the temperature will decrease the complete permeation of both Ar and Kr, with a lower decrease for Kr. This is attributed to the fact that at lower total number densities, systems reach equilibrium conditions at a much faster rate than at higher number densities, impeding further permeation. Figure 17a–d show the significant decrease in Kr occupancy in inner cages but not in the complete permeation region. The density profiles in Figure 17e,f show the effect of a temperature increase to 425 K on the atom numbers along the direction of permeation, where changes inside the membrane mainly affect Kr, and changes at the complete permeation region mainly affect Ar.

#### 3.2.5. Mixtures of Xenon and Argon

**Figure 18:**
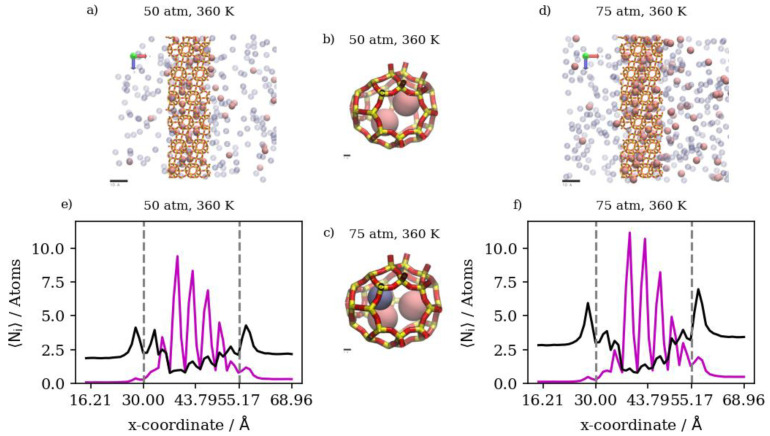
(**a**,**d**) An orthographic view of the adsorption layer adjacent to the membrane, (**b**,**c**) schematic of an alpha cage in the membrane, and (**e**,**f**) density profiles of pressure’s effect on Xe/Ar gas mixture in DD3R at 360 K. Atoms’ colors: Xe (pink), Ar (violet). Density profiles’ colors: Xe (magenta), Ar (black).

In a similar gas mixture of Xe/Ar at a 10:90 ratio, the numerical results show that changes in pressure and temperature have limited effects on Xe’s complete permeation. This is attributed to the barrier-restricted diffusion of Xe; the larger atom size of Xe relative to the window dimensions would require unrealistically long simulation times to observe complete permeation. Carrying out GCMC prior to permeation MD permits us to approach equilibrium Xe occupancies in the membrane region at various conditions of pressure and temperature, thus providing the proper conditions for Ar adsorption and permeation in the presence of Xe. However, the Xe permeation MD is still sterically limited by the atomic size of Xe. The GCMC simulations permit us to observe the effects of changes in pressure and temperature in the membrane region. We note that increases in the retentate are less pronounced at 360 K than at 425 K, due to the system approaching maximum capacity of the inner cages at lower pressure values at the former temperature. On the other hand, the changes in Xe’s complete permeation are within the range of statistical errors; the full realization of quantitative results would require unrealistically long simulation times. Nevertheless, the available results do show trends that indicate complete permeation tending to increase with increases in pressure, and tending to decrease with increases in temperature. For Ar in Xe–Ar mixtures, complete permeation and membrane content increase with increasing pressure at a constant temperature in large numbers compared to Xe, due to its smaller size. It is worthy to note that adsorption selectivity is highly favorable toward Xe in this case, where the ratio of Xe/Ar in the membrane region is 1.5, unlike in the Kr/Ar simulation, where the ratio is 0.5. Increasing the temperature has the opposite effect, in which the amounts of adsorbed gas atoms on the membrane surface as well as the amount of completely permeating atoms decrease. The effect of temperature on Xe behavior in the retentate region is more obvious than the pressure effect, where the occupancy of inner cages is reduced at elevated temperatures, as expected for an adsorption-dominated permeation. The molecular visualizations in Figure 18a–d show the effects of pressure on permeation in a Xe–Ar mixture. Xe occupancy is determined by the GCMC simulation and hardly changes during the permeation MD; thus, it is not highly affected by the increase in pressure, unlike Ar, as additional Ar atoms can occupy cages with Xe at the maximum capacity of two atoms, according to the experimental data available in the literature. The calculations from density profiles of Ar and Xe illustrated in Figure 18e,f show that the integral of the density profile within the membrane boundaries only increases by 20 percent when the pressure is increased from 50 atm to 75 atm at 360 K, unlike the case of the adsorption layer at the membrane’s surface, where the increase is equivalent to 55 percent.

**Figure 19:**
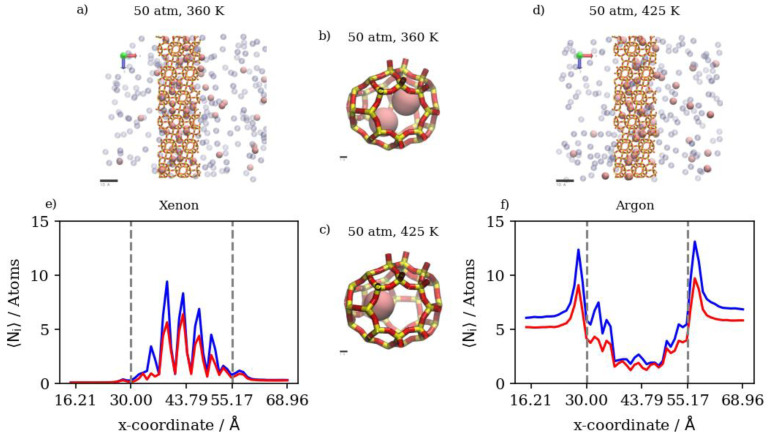
(**a**,**d**) An orthographic view of the adsorption layer adjacent to the membrane, (**b**,**c**) schematic of an alpha cage in the membrane, and (**e**,**f**) density profiles of temperature’s effect on Xe/Ar gas mixture in DD3R at 50 atm. Atoms’ colors: Xe (pink), Ar (violet). Density profiles’ colors: 360 K (blue), 425 (red) (see Figure 6 for molecular level picture description).

**Figure 20:**
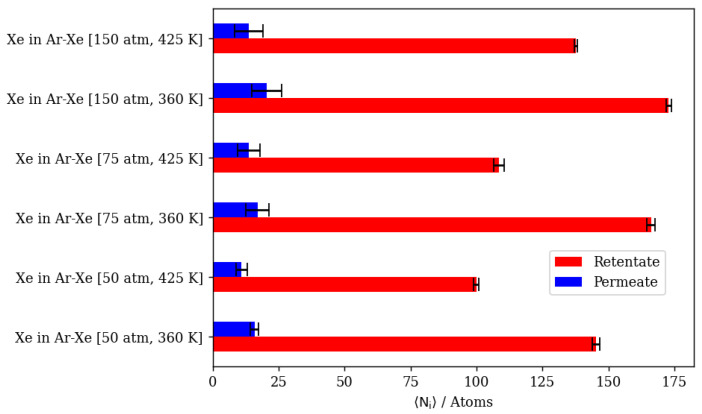
Xe permeance through DD3R zeolite at different temperatures and pressures.

The effects of temperature change on Xe/Ar occupancies and the adsorption layer are shown in Figure 19a–d. The increasing temperature decreases the Xe occupancy of inner cages of DD3R during the GCMC simulation more so than Ar, which does not occupy inner cages in high numbers in the presence of Xe. The density profiles in Figure 19e,f show the effects of temperature on Xe in the inner cages and the effects on Ar at the sides of the membrane slabs. Figure 20 summarizes the numerical results from counting the atoms in both the retentate and permeate regions of the simulation box for Xe/Ar simulations. Although the permeation of Xe is barrier-limited and complete permeation is essentially statistically insignificant for the lengths of our simulations, the trends indicate Xe permeation tending to increase with increases in pressure, and tending to decrease with increases in temperature.

#### 3.2.6. Ternary Mixture: Krypton, Xenon, and Argon

Here we compare the results of simulations of equimolar Kr/Xe mixtures in Ar at a 5:5:90 ratio with the results of Kr/Ar and Xe/Ar simulations, again keeping in mind that for Xe, GCMC simulations provide the adsorption and inner cage occupancies, but the permeation MD step that follows does not yield statistically significant results and only shows trends for Xe permeation, due to barrier-limited cage-to-cage dynamics. In the case of the equimolar Kr/Xe mixture with Ar, competitive adsorption causes the complete permeation of both Kr and Xe to decrease in comparison to Kr/Ar and Xe/Ar simulations.

**Figure 21:**
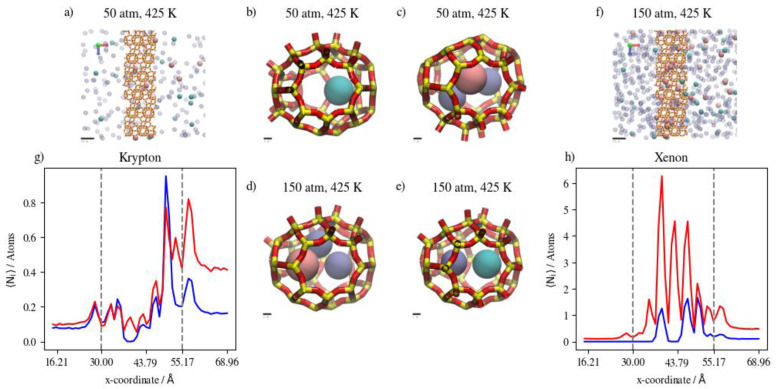
(**a**,**f**) An orthographic view of the adsorption layer adjacent to the membrane, (**b**–**e**) schematic of an alpha cage in the membrane, and (**g**,**h**) density profiles of pressure’s effect on Kr/Xe/Ar gas mixture permeation at 425 K. Atoms’ colors: Kr (cyan) Xe (pink), Ar (violet). Density profiles’ colors: 50 atm (blue), 150 atm (red) (see Figure 6 for molecular level picture description).

Increasing the pressure at a constant temperature of 360 K increases the complete permeation of Kr and decreases that of Xe. At 425 K, this increase in Kr permeation is limited by the slight increase in Xe complete permeation when the pressure is increased. This is expected for Xe, as it needs the higher temperature to be able to overcome the energy barrier associated with the process of hopping into and out of DD3R alpha cages. Figure 21a–f show a molecular visualization of the effects of increasing pressure at 425 K, where the Xe and Ar occupancy of inner cages is increased, but not that of Kr. In fact, the impeding of Kr permeation is visible in Figure 21f,g where more Kr atoms remain in the feed region due to the saturation of the membrane structure with Xe, and to a lesser extent with Ar. The density profiles showing the effects of pressure on Kr/Xe with Ar sweep gas permeation in Figure 21g,h illustrate the limited impact of a pressure increase at high temperature conditions on Kr and Xe permeation. The Kr density profiles show very limited changes in the membrane and complete permeation regions. Higher pressures and temperatures have an obvious effect on the increase in Xe content in both regions. At the adsorption layer, the Kr/Xe ratio goes down from 1.45 to 0.73 when pressure is increased from 50 atm to 150 atm at 425 K. This causes permeation selectivity to change from infinite selectivity in favor of Kr to 0.91 for Kr/Xe, showing the effect of the higher driving force on Xe permeation at higher temperatures.

**Figure 22:**
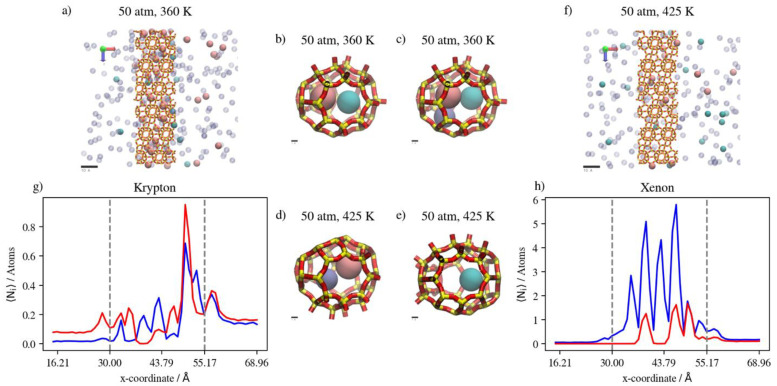
(**a**,**f**) An orthographic view of the adsorption layer adjacent to the membrane, (**b**–**e**) schematic of an alpha cage in the membrane, and (**g**,**h**) density profiles of temperature’s effect on Kr/Xe/Ar gas mixture permeation at 50 atm. Atoms’ colors: Kr (cyan) Xe (pink), Ar (violet). Density profiles’ colors: 360 K (blue), 425 K (red) (see Figure 6 for molecular level picture description).

Increasing the temperature at a constant pressure of 50 atm reduces the occupancy of the inner cages by gas atoms. This is expected for an adsorption-dominant permeation. The higher temperature yields higher Kr complete permeation at 50 atm, unlike Xe, which decreases the occupancy of inner cages significantly. This is a direct consequence of the higher Kr/Xe ratio at the adsorption layer being larger (1.45) at the higher temperature in comparison to the lower temperature, where the ratio is lower (0.65). Figure 22a–f shows the molecular visualization of the effect of increasing temperature from 360 K to 425 K at 50 atm. The overall cage occupancy is reduced at the higher temperature, causing Kr to permeate at a much faster rate given the limited number of available adsorption sites. Density profiles in Figure 22g,h show the limited impacts of temperature change on the Kr density profile within the membrane and the complete permeation regions, unlike Xe, which is significantly impacted. We note once again that Xe’s hopping into alpha cages is barrier-height-limited, and our MD simulations do not capture this behavior. In other words, the reduced occupancy of Xe at these conditions is attributed to the GCMC insertion simulations resulting in a prefilled membrane structure, which we have verified to have reached equilibrium in all simulations considered in the previous comparisons.

**Figure 23:**
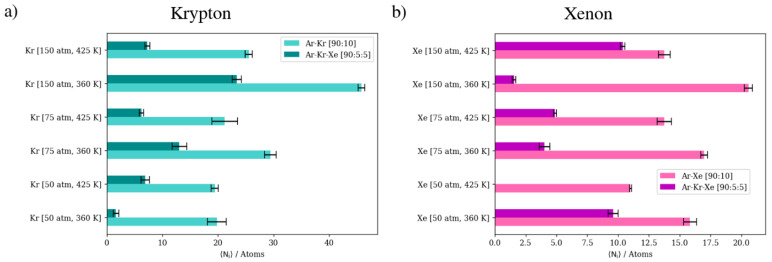
Complete permeation of gas atoms through DD3R zeolite at different temperatures and pressures with Ar as the sweep gas. (**a**) Kr permeation, (**b**) Xe permeation.

The summary of numerical results in Figure 23 suggests that higher pressures and lower temperatures are preferential for high Kr/Xe separation when Ar is present as the sweep gas. At 150 atm and 360 K, the Kr/Xe separation factor is 22. However, infinite separation selectivity in favor of Kr was observed at 50 atm 425 K. Nonetheless, this infinite selectivity comes at the cost of permeation throughput, which decreases due to the inhibition of adsorption at elevated temperatures. The direct numerical comparison shows that the complete permeation of Kr at this infinite selectivity at 50 atm and 425 K is a third that of what it is at 150 atm and 360 K.

#### 3.2.7. Diffusion in Pure Gases and Mixtures, Temperature, and Pressure Dependence

**Figure 24:**
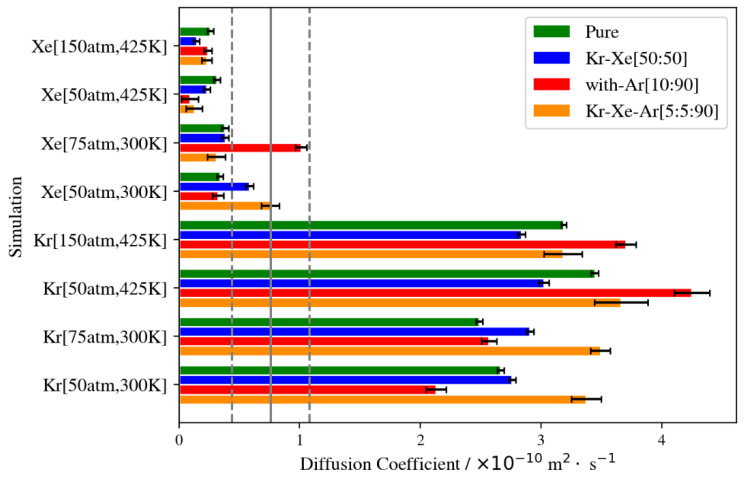
Kr/Xe MSD diffusion coefficients at different conditions. Solid gray line shows experimental Kr diffusivity as pre-exponential in DD3R. The dashed lines correspond to the errors associated with the experimental data [12].

Here we show the results of mean squared displacement (MSD) diffusion. We note that we are only comparing the Kr diffusion coefficient obtained from our MD to experimental diffusion results available in the literature. The available results were derived from experiments carried out at 298 K, a much lower pressure range, and using thicker membranes (5 µm). Therefore, only an order of magnitude comparison would make sense with such differences in the approach to diffusivity measurement. As shown in Figure 24, the experimental diffusivity pre-exponential value is (0.76 ± 0.32) × 10^−10^ m^2^/s, which is the same order of magnitude as in our MSD diffusion results [12]. The higher results are expected from MD gas permeation simulations because of the higher pressure required for quantitative analysis, which puts simulation systems in non-equilibrium states, causing diffusion coefficients to incorporate a drift component due to the high-pressure gradient across the membrane slabs. This has been discussed as part of comparisons of experimental diffusivity results to simulation results in the literature [28]. Defect formation during membrane synthesis may contribute to such discrepancies, in addition to previously discussed reasons, such as differences in membrane thickness, operating temperature or pressure.

The summary of MSD diffusion illustrated in Figure 24 shows the effects of the different conditions of the gas permeation simulations. Increasing the pressure at the lower temperature increases the diffusion coefficient of Kr when it is presented with Xe, Ar or both. This is not the case for pure Kr, where increasing pressure from 50 atm to 75 atm at a constant temperature of 300 K decreases the diffusion coefficient due to the blockage of the membrane channel system, as multilayer absorption takes place at the surface exposed to the gas feed, as explained earlier in pure Kr simulations’ density profiles. This trend is reversed when pressure is increased at the higher temperature, in that the Kr diffusion coefficient decreases when it is present with other gas species. This is caused by the limited number of active adsorption sites available for Kr atoms at the higher temperature. In such a case of competitive adsorption, adsorption selectivity is higher for Xe due to its greater interaction with the membrane framework, and for Ar, due to its much higher partial pressure. These reasons contribute to the decrease in Kr diffusivity when pressure is increased at 425 K. Increasing the temperature at a constant pressure, on the other hand, increases the Kr diffusion coefficient for all gas feed compositions. This is attributed to the higher kinetic energy at the higher temperature and the reduced capacity of inner cages of the membrane, causing Kr to pass through the DD3R structure at faster rates than at lower temperatures. Given the lack of experimental data concerning Xe diffusion in DD3R and the fact that our MD simulations do not capture the full scope of Xe permeation, given its larger size and the prohibitively long simulation times needed, the quantitative analysis of Xe diffusivity will be inconclusive. Nonetheless, the comparison of Xe results to Kr results reflects the experimental separation trends, where Xe diffusivity drops when present in a mixture with Kr, whereas Kr diffusivity goes up under the same conditions.

## 4. Discussion

While the GCMC experiments show reasonable results for pure gas adsorption, as shown by the resulting adsorption isotherms shown in Figure 4, not all mixture gas runs reach equilibrium, and achieving true adsorption equilibrium would require prohibitively long simulation times. Nonetheless, all runs were trending the right way toward equilibrium, under which conditions adsorption selectivity favors Xe more than both Kr and Ar. Within the number of GCMC exchanges considered for all runs, simulations at low pressure and high temperature reached equilibrium, making them utile in studying how pressure, temperature, and gas composition affect inner cage capacity and its occupancy with different gas atoms, and ultimately gas permeation.

Our MD simulations show how the adsorption-dominated permeation of gas through DD3R is extremely sensitive to changes in pressure, temperature, and composition. Having near-equilibrium adsorption conditions has also been proven critical for studying separation selectivity, as the adsorbed phase compositions show direct effects on gas permeation. The Kr/Xe ratio in the adsorbed phase is strongly correlated with permeation selectivity, in that the lower the Kr/Xe ratio in the membrane region, the higher the Kr permeation, provided the presence of a Kr driving force sufficient for completely passing through the membrane structure. The higher partial pressure of Kr provides such a driving force that enhances adsorption until the saturation of the adsorption monolayer is reached at the surface exposed to the gas feed, which impedes further Kr permeation and causes multi-layer adsorption. Increasing the temperature reduces the adsorption layer’s capacity and increases the kinetic energy of gas atoms, facilitating higher fractional permeation. This effect of temperature is also limited by the fact that a higher temperature causes density values to plummet. Therefore, a continuous membrane-based separation would be a reasonable approach for the separation of Kr/Xe mixtures under these conditions.

The presence of Ar plays an important role in promoting higher Kr permeation, which is normalized to the total number of Kr atoms in each system. This was shown by the fact of Kr/Xe separation selectivity increasing when Ar was introduced in most of our gas permeation runs. This increase was not observed when temperature was increased due to the very low Kr density numbers at elevated temperatures.

## 5. Conclusions

In this work, we observed experimental trends for Kr/Xe separation via use of the DD3R zeolitic membrane. Our studies show that the permeation of pure and equimolar gas feed increases with increasing pressure and decreases with increasing temperature. In addition, the permeation of Kr increases when in an equimolar mixture with Xe, while the latter increases its surface adsorption due to a higher adsorption selectivity. Observations of the molecular trajectories show that a state of inner cage occupancy that is saturated with respect to Xe but not Kr is preferable for higher Kr permeation. In our previous paper, we showed that the presence of Xe inside the inner cages of the membrane was critical in reproducing experimental trends wherein the Kr/Xe separation ratio was high in favor of Kr by using MD simulations [13]. Artificially filling all the inner cages with the maximum occupancy of Xe was sufficient to show the significant effects of Kr–Xe attractive forces on promoting higher Kr complete permeation. In this work, GCMC insertion simulations were used to introduce adsorption equilibrium conditions into the inner cages of the membrane slabs used in our MD simulations, giving a realistic representation of adsorption equilibrium conditions at different pressures and temperatures. The use of Ar as the sweep gas, as is done in other experimental works on such separations, causes both gases to increase their adsorption, with higher Kr permeation in comparison with pure Kr gas. We conclude that Kr–Xe attractive forces contribute to the higher Kr permeation shown in our previous paper, but this is only one factor among many others. The adsorbed phase composition and total driving force (pressure and composition differences) are also equally important. However, if the pressure difference is very large, then in some cases, the cages get blocked by the Xenon adhering to the cage openings. Given the sensitivity of gas separation to such different conditions, we undertook a screening of the effects that the different conditions have on the density profiles of different gas species along the gas permeation direction, along with quantitative comparisons of the permeation throughput and the diffusion coefficients of Kr and Xe. All these findings play an important role when designing separation unit operations for the Kr/Xe off-gas byproducts emitted at contemporary nuclear power plant installations.

## Figures and Tables

**Table 1:** 12-6 Lennard–Jones potential parameters.

Pair	*ε* (kcal/mol)	*σ* (Å)	Reference
Si–Si _(zeolite)_	0.0010	1.000	[8]
O–O _(zeolite)_	0.1898	3.000	[8]
Ar–Ar _(gas)_	0.2380	3.405	[23]
Kr–Kr _(gas)_	0.3380	3.690	[23]
Xe–Xe _(gas)_	0.4190	4.100	[23]

## Data Availability

The data that support the findings of this study are available from the corresponding author upon reasonable request.
